# It is time for more holistic practices in mental health

**DOI:** 10.1371/journal.pmen.0000028

**Published:** 2024-06-04

**Authors:** Sidarta Ribeiro, Ana P. Pimentel, Valter R. Fernandes, Andrea C. Deslandes, Paulo Amarante

**Affiliations:** 1 Brain Institute, Federal University of Rio Grande do Norte (UFRN), Natal, Brazil; 2 Psychiatry Working Group of the Centre for Strategic Studies, Fundação Oswaldo Cruz (FIOCRUZ), Rio de Janeiro, Brazil; 3 Laboratory of the Neuroscience of Exercise, Institute of Psychiatry, Federal University of Rio de Janeiro (UFRJ), Rio de Janeiro, Brazil; PLOS: Public Library of Science, UNITED KINGDOM

Hegemonic psychiatric models in the 20^th^ century, centered on the notion of disease, were willing to consider a single ’natural’ primary cause—either biochemical or neurophysiological–as sufficient explanation for complex psychological and social phenomena like psychosis or depression. The pharmacological treatment of mental health conditions came to be understood solely in terms of metabolic replenishment to fight a putative ’chemical imbalance’, without a true need to delve into the recesses of the patients’ minds and bodies.

These overly simplistic models collapsed in the past two decades, and the trend is now the opposite, towards the recognition that mental health conditions are to a large extent, a social construct produced by lifestyles that jeopardize the essential tenets of good health: sleep, nutrition, exercise, introspection, and mind-body connection. Although psychiatric drugs are widely prescribed as ’effective’ treatment, the number of people diagnosed with mental health conditions has increased substantially in the past decades. The World Health Organization (WHO) estimates that the prevalence of mental health conditions has risen from 416 million people in 1990 to over 615 million people in recent years [1]. Similarly, the U.S. National Survey on Drug Use and Health has reported significant increases in rates of depression, anxiety, and suicidal ideation, particularly among young adults, over the past decade [2]. A significant increase in the diagnosis of attention deficit hyperactivity disorder (ADHD) in children has also been reported in the U.S. [3] and other countries. These increases have been attributed to better diagnosis and the increasing prevalence of conditions like anxiety and depression, which likely reflect the negative impact of contemporary lifestyle on mental health. Nevertheless, two other factors often overlooked, may also contribute to explain increase: the iatrogenic effects of psychotropic drugs, and overdiagnosis due to financial conflicts of interest, or prejudice against neurodivergence, as may be the case of ADHD and certain forms of autism.

Like Thomas Insel, head of the National Institute of Mental Health for 13 years, publicly acknowledged during the 2017 Psychedelic Science meeting in Oakland, even though psychiatrists feel that they have come a long way in recent decades, patients are drowning in anxiety, depression, despair, and suicide. At the same time, there is a growing interest in traditional holistic practices and therapies, from Yoga, Qigong, and Capoeira, to gardening, meditation, and dreamwork. What is going on?

## Medicalization should be reduced

How can this situation be changed? First, psychiatry must reduce medicalization as a commitment to doing no harm, by engaging in the multidimensional construction of alternatives to the conventional paradigm, which despite being dominant, is ineffective in the face of the complex challenges that involve mental care, such as stigma, social exclusion, and violation of rights. Medicalization reduction works not with the concept of ’disease’, which explains the processes related to health-suffering-care solely in biological, chemical, and physical terms. Instead, it seeks to broaden the understanding of these processes through the dialogue with different sources of knowledge to create therapeutic environments capable of favoring the development of emancipation and autonomy, increasing the possibilities of the person in care to act on the problems and challenges that arise in life without resourcing to mandatory medical treatment, making use of health services only when necessary and focusing on the active participation of the subject in the treatment. Importantly, this approach works with the ’biographical perspective’, a fundamental principle related to the understanding that each person has a story to tell, which expresses the knowledge arising from their own experience.

In its most recent global report on mental health, the World Health Organization (WHO) drew attention to the urgency of transforming mental health care towards providing care based on respect for the rights and dignity of the people assisted: “*Around the world*, *mental health needs are considerable*, *but responses are insufficient and inadequate*” [1]. An important aspect of the report is the emphasis placed on the importance of lived experience and the presentation of successful stories of medicalization reduction, which include many successful practices documented across several countries from the 1970s onwards, in the wake of the Psychiatric Reform. Consolidated examples of crisis care in Mental Health include the ‘Open Dialogue’ approach [4], the ‘Soteria Houses’ [5], and peer-run nursing homes such as ‘Peer Respite’ [6], which have contributed to the reduction of chronicity, disabilities, and other harmful effects of the conventional ways of treating acute crises and psychic emergencies. Particularly interesting are the practices based on peer support, i.e., on mutual support between people who go through similar health distress and have the objective of enhancing the overcoming of the difficulties brought about by the experience of mental suffering through a relationship of equality, trust, and mutual respect between the participants of the group, i.e., the peers. The individual´s environmental and social setting seems to be the first target that should be addressed in any kind of treatment for mental health.

## Mens sana in corpore sano

There must be a change of focus from ‘disease’ to ‘healthy lifestyle’. To a large extent, rather than chemical imbalance in isolation, it is the physiological, hormonal, and psychological imbalance caused by poor sleep, inadequate nutrition, and scant exercise that snowballs into severe individual and social suffering. Psychiatry and other health-related specialties must engage in a consistent effort to rescue the basic tenets of health. There is abundant evidence that poor sleep increases the risks for a plethora of problems ranging from cognitive and emotional impairment to anxiety, depression and withdrawal of human helping [7]. Fixing bedtime habits to promote better sleep is a simple yet critical step towards improving mental health.

Adequate nutrition is another key factor that often gets overlooked in the pursuit of better mental health. Unhealthy diets based on ultra-processed products, lacking in fibers and fermented food, and contaminated with pesticides and fertilizers, can decrease the microbiome diversity, promote body inflammation, hinder the immune response and lead to various disorders, including anxiety and depression [8]. It is urgent to incorporate non-processed food, rich in fiber and fermented ingredients, into standard diets across the globe. It is also urgent to end the starvation that reaches nearly 10% of the world’s population.

Regular suitable exercise is the third essential aspect of mental health that often gets neglected. Sedentary lifestyles with little physical activity and much recreational screen time are associated to various mental health risks [9]. Passive behaviors like watching television have been linked to an increased risk of depression [10]. Fortunately, even a few minutes per day of moderate to vigorous exercise can have a major positive impact on cognition, mood, and overall health. In particular, non-Western integrative practices such as Yoga and Capoeira show promise to improve cognition, mood and overall well-being [11,12]. Beyond the benefits rooted in the exercise´s characteristics and physiological demands, their practitioners embrace them as a philosophy of life, fostering a sense of belonging and community that promotes a healthy mental environment.

## Inner dialogue is necessary

A dialogue with the more visceral and subjective aspects of the mind is crucial for a healthy life, including the key role played by sexuality, and creative activities such as active imagination. A deeper engagement with proprioception, interoception and introspection can greatly catalyze healing processes, broadening self-understanding, and improving well-being. Non-iatrogenic activities such as nature exposure [13], art therapy [14], and gardening [15] are protective against mental suffering. These expressive outlets offer unique, personalized paths towards healing and self-discovery, offering a holistic and integrated approach to psychiatry.

Likewise, the dialogue with discourse-based depth psychology, interrupted for nearly a century, should be resumed. Psychotherapy has proved itself not just as clinically effective, but also essential for treatment of most psychiatric cases [16]. The quantitative structural analysis of patients’ dreams can offer profound insight into their mental state, as shown to be the case for the early diagnosis of schizophrenia [17]. The analysis of the emotional content of dream reports, in particular nightmares, can provide valuable insight into different neurological conditions [18]. Furthermore, automated speech analysis through machine learning can be successfully used to detect and track emotions over time [19]. The emotional relevance of dreaming and the clinical effectiveness of talk therapy underscore the need for a multidimensional approach to mental health care, capable of listening attentively to the patients. Discourse analysis at the structural and symbolic levels gives introspective access to deeper layers of the psyche, may help to build the sense of meaning in life, and must be more widely integrated into treatment plans.

## Psychiatry must dream a better society

Mental health conditions are often caused by physiological imbalance that is socially constructed. Deficits in sleep, nutrition, exercise, introspection, and other pillars of good mental health do not occur in the vacuum, they are produced by how we live. Precarious working and housing conditions, unemployment, racism, misogyny, homophobia, transphobia, physical and symbolic violence, the war on people that use certain drugs, and other forms of social bias all contribute to mental suffering and need to be considered systemically. The individual’s environmental and social setting must be understood and improved through integrative practices. It is time to strive towards a more naturalistic and benign approach to promoting mental well-being, by strengthening the connections to one’s own body, nature, and community.
